# An empirical evaluation of kernels for time series

**DOI:** 10.1007/s10462-021-10050-y

**Published:** 2021-07-27

**Authors:** Mourtadha Badiane, Pádraig Cunningham

**Affiliations:** School of Computer Science, University College Dublin, Dublin, Ireland

**Keywords:** Time-series classification, Support vector machines, Kernel methods

## Abstract

There exist a variety of distance measures which operate on time series kernels. The objective of this article is to compare those distance measures in a support vector machine setting. A support vector machine is a state-of-the-art classifier for static (non-time series) datasets and usually outperforms k-Nearest Neighbour, however it is often noted that that 1-NN DTW is a robust baseline for time-series classification. Through a collection of experiments we determine that the most effective distance measure is Dynamic Time Warping and the most effective classifier is kNN. However, a surprising result is that the pairing of kNN and DTW is not the most effective model. Instead we have discovered via experimentation that Dynamic Time Warping paired with the Gaussian Support Vector Machine is the most accurate time series classifier. Finally, with good reason we recommend a slightly inferior (in terms of accuracy) model Time Warp Edit Distance paired with the Gaussian Support Vector Machine as it has a better theoretical basis. We also discuss the reduction in computational cost achieved by using a Support Vector Machine, finding that the Negative Kernel paired with the Dynamic Time Warping distance produces the greatest reduction in computational cost.

## Introduction

In this paper we aim to determine the best classifier for time series by experiments conducted on datasets freely available from the UCR repository. We re-farmiliarize the reader with five time series distance measures: Dynamic Time Warping, Edit Distance on Real Sequences, Time Warp Edit Distance, Symbolic Aggregate Approximation, and Symbolic Fourier Approximation. Then we present the results of a kNN and SVM classifier paired with each distance measure and evaluated on each dataset. We find that the Gaussian Support Vector Machine paired with Dynamic Time Warping distance is the most accurate time series classifier. We also find that a significant reduction in computational cost can be found by using the Negative Support Vector Machine paired with the Dynamic Time Warping distance.

### Recapitulation

Support Vector Machines have a well deserved reputation as one of the best classifier methods available. SVMs became popular in the 1990s and many powerful implementations have come available in recent years (Boser et al. 1992; Cortes and Vapnik 1995).

SVMs can classify data where the classes are not linearly separable by mapping the data into a higher dimension feature space where the classes are linearly separable. This mapping is achieved using a kernel function that is applied to each pair of data points in turn to produce a kernel matrix. If the kernel matrix is positive semi-definite (PSD) then training the SVM is a convex optimization problem and an optimum solution can be found. However, if this kernel matrix is not PSD then the optimality of the SVM training process is not guaranteed.

If the data follows a standard feature vector representation then there are standard kernels available that are PSD and will produce effective classifiers. For a kernel to truly be a kernel in the strictest sense it most correspond to an inner product in it’s Hilbert reproducing space, this highly mathematical criterion simplifies to a condition known as positive semi-definiteness. This means that the Gaussian kernel must be paired with a distance measure that is a metric i.e. it must satisfy positive definiteness, symmetry, and the triangle inequality. It is known that the distance measure derived from the following three algorithms do not satisfy the triangle inequality:

- Dynamic Time Warping (DTW) (Keogh et al. 2001),
- Symbolic Aggregate Approximation (SAX) (with edit distance) (Lin et al. 2003),
- Symbolic Fourier Approximation (SFA) (with edit distance) (Schäfer and Högqvist 2012).

It is perhaps worth noting that it is trivial to recognise the latter two cannot be metrics since they are built upon edit distance which is not a metric. The researcher who wishes to use SVMs for time-series classification has a few options:

DTW with 1 Nearest Neighbour (1NN) is considered an excellent base-line classifier for time-series (Bagnall et al. 2017), this paragraph acts as a brief digression to explain this before we continue with our analysis of kernel methods below. With SVM classification we must chose a kernel which should act like an inner product in a feature space. Distinct kernels which preserve order i.e. $k_{1}(x,y) \le k_{1}(w,z) \iff k_{2}(x,y) \le k_{2}(w,z)$ will lead to completely different SVM classifiers, the same cannot be said about kNN. For example the Gaussian kernel $e^{-x}$ and the negative kernel $-x$ both reverse and then preserve the order. In other words if $e^{-a} \le e^{-b} \iff -a \le -b$. One possible reason why kNN performs better than SVMs in time series classification is that kNN has fewer parameters to tune, and every function of the distance will produce identical results in kNN so long as it is order preserving. This is not true for SVMs, if we have some distance ordering then we can form two distinct similarity measures: the negative kernel and the Guassian kernel. This added parameter: the choice of kernels means that kNN is simpler than SVMs and is perhaps one reason for its dominance in time series analysis.

Another possible reason which these authors intend to explore in later publications is that kNN is superior to SVM because all the time series studied are univariate. Upon careful consideration one can clearly imagine that this is most definitely the case, since kNN is always going to be superior in a one dimension space for datasets that are not composed of time series.

## Time series classification with support vector machines

### Problem setting

Time series classification involves training a classifier on time series and using that classifier to predict the target of a novel sample. Firstly, to allow for brevity in explanation we make the definition $[k] = \{1,...,k\}$. We are given N training samples $\{x^{(i)}\}_{i=1}^{N}$ where each $x_{i}$ is an ordered set of vectors, called a time series, of (perhaps varying) length $m_{i}$, i.e. $x^{(i)} = \{x^{(i)}_{j}\}_{j=1}^{m_{i}}=\{x^{(i)}_{1},...,x^{(i)}_{m_{i}}\}$ where $x^{(i)}_{j} \in {\mathbb {R}}^{D} \quad \forall i \in [N] \quad \forall j \in [m_{i}]$. D is the spatial dimensionality of the problem setting and is the same for all sample time series. Lastly, associated with each sample $x^{(i)}$ is a target class either $+1$ or $-1$. The problem is then: given the training data, a collection of time series $\{x^{(i)}\}_{i=1}^{N}$ and class labels $y_{1},...,y_{N}$ build a function which maps novel time series to $\pm 1$ that best approximates the true hypothesis function which maps the data to their correct classes. For multi-class classification we have an identical problem setting except that the class labels of the variables are not constrained to $\pm 1$, they can take on any number of discrete class labels.

### Support vector machines

The support vector machine (SVM) is a state-of-the-art classifier that works by constructing a hyperplane that best fits the data. Without using a non-linear mapping, the algorithm works by solving a quadratic programming problem which maximizes the margin between the two classes where the margin is the space between the two class borders. Unfortunately this simple approach cannot be used to correctly classify data that is not linearly separable. To overcome this flaw support vector machines make use of a map which sends the data to a higher dimensional feature space where the data is linearly separable. The separator is a hyperplane in the feature space but not necessarily in the input space. When using SVMs we do not actually need to know the feature map instead it is enough to know only the inner product between feature vectors. This inner product on feature vectors is called the kernel of an SVM. An SVM is only as good as the kernel it uses. The criterion for a function to be a kernel is that it must define an inner product in some feature space.

For a detailed and thorough explanation of the training and prediction procedure of support vector machine written by the discoverer Vladimimr Vapnik please see (Cortes and Vapnik 1995).

### Positive semi-definiteness

As stated earlier we require the function we are using as a kernel to behave like an inner product in the feature space. It has been shown that for a function to behave like an inner product in a feature space it is necessary and sufficient that it be positive semi-definite (PSD) (Badiane et al. 2018). A function which is not PSD is known as indefinite. A function $K \in {\mathbb {R}}^{D \times D}$ is PSD if $x \cdot Kx \ge 0 \quad \forall x \in {\mathbb {R}}^{D}$. It is trivial to show (and its in the paper last cited) that a kernel matrix is PSD if all its eigenvalues are non-negative. It is known that the SVM optimization process is a convex cone problem with a global optimal solution when the kernel is PSD, however when the kernel is not PSD then the SVM optimization process may terminate at a local optimum distinct from the global optimum. This sub-optimal performance has led many to attempt to tackle the problem of indefinite kernels.

## Distance measures for time series

This section provides some detail on the three measures (DTW, SAX and SFA). It explains how they work and illustrates the problem that they do not readily produce PSD kernels. Once we posses a distance measure *d* on time series *x* and *y* we can form the kernel in one of two obvious ways:

- The Negative kernel: $K(x,y) = -d(x,y)$.
- The Gaussian kernel: $K(x,y) = e^{- \gamma d(x,y)}$.

$\gamma$ is a parameter to be optimized.

### Dynamic time warping

We will re-introduce the DTW algorithm presented in (Shimodaira et al. 2002) as well as (Cuturi 2011) and (Cuturi et al. 2007) . We begin this section by the definition of an alignment between two time series. We then define the DTW kernel in terms of those alignments. Both these definitions were made in (Shimodaira et al. 2002).

**Fig. 1:**
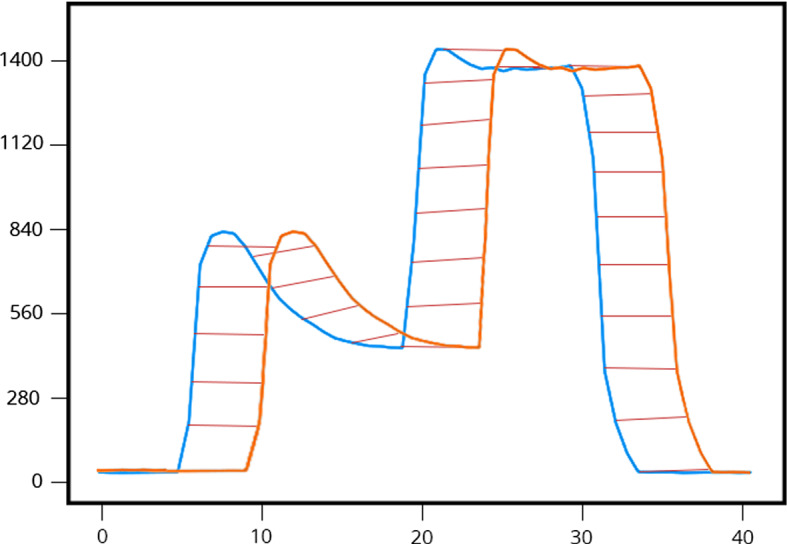
Misalignment correction by DTW (see Mahato et al. (2018))

To compare two time series that do not line up on the *x*-axis we may use the dynamic time warping distance. As you can see in Fig. 1 when comparing two time series *X* and *Y* that are similar in shape except that the crest of one is shifted along the *x*-axis, the DTW kernel will reflect this similarity by warping the time axis so that the two time series align. In contrast the Euclidean distance completely ignores the inherent similarity between the two series as a result of the misalignment. In summary DTW is elastic, and Euclidean distance is not.

The dynamic time warping distance is not a metric since it does not satisfy the triangle inequality^1^ If the distance underlying a kernel fails this test, the kernel will fail to be PSD. A kernel that is not PSD is known as indefinite, and in theory the SVM optimization process involved when using an indefinite kernel need not be a convex quadratic programming problem, as a result the training algorithm is not guaranteed to terminate at a global optimum. This theoretical problem turns out to be of little consequence in experimentation, as we find in the experiments section that very often there is only a tiny margin in difference between the Gaussian and Negative kernel even though the former nearly always produces PSD kernels while the latter does not. For example with DTW there is no (statistically significant) difference in mean accuracy between the Gaussian and the Negative Kernels.

To calculate the dynamic time warping distance we must first define what is meant by a good alignment. An alignment $\pi$ is a pair of functions $\pi ^{1}$ and $\pi ^{2}$ which satisfy the following properties: ($[v] = \{1,...,v\}$)

1 $$\begin{aligned}&\pi ^{1}: [m] \rightarrow [l]\end{aligned}$$

2 $$\begin{aligned}&\pi ^{2}: [n] \rightarrow [l] \end{aligned}$$

where *l* is known as the length of the alignment.

3 $$\begin{aligned} \pi ^{k}_{1} = 1,\quad for \quad k \in [2] \end{aligned}$$

and

4 $$\begin{aligned}&\pi ^{1}_{l} = m \end{aligned}$$

5 $$\begin{aligned}&\pi ^{2}_{l} = n \end{aligned}$$

6 $$\begin{aligned}&\pi ^{k}_{i}-\pi ^{k}_{i-1} \in \{0,1\} \quad \forall k \in [2] \ \forall i \in \{2,...,l\} \end{aligned}$$

7 $$\begin{aligned}&\pi ^{a}_{i}=\pi ^{a}_{i-1} \implies \pi ^{b}_{i} - \pi ^{b}_{i-1} = 1, \ \forall a,b \in [2], a \ne b, \ \forall i \in \{2,...,l\} \end{aligned}$$

We may summarize the criteria as both $\pi ^{1}$ and $\pi ^{2}$ must be monotonic functions from [*m*] and [*n*] onto [*l*] such that they contain no simultaneous repetitions (11).

Once we have the alignment $\pi$ we may define the dynamic time warping distance between two time series ${\mathbf {x}}$ of length *m* and ${\mathbf {y}}$ of length *n*.

8 $$\begin{aligned} d({\mathbf {x}},{\mathbf {y}}) = \min \limits _{\pi \in {\mathcal {A}}({\mathbf {x}},{\mathbf {y}})}(\sum _{k=1}^{l}\Vert {\mathbf {x}}(t_{\pi ^{1}_{k}}) - {\mathbf {y}}(t_{\pi ^{2}_{k}}) \Vert ) \end{aligned}$$

where ${\mathcal {A}}({\mathbf {x}},{\mathbf {y}})$ is the set of all possible alignments and $\Vert . \Vert$ is the regular Euclidean distance.

We may calculate the dynamic time warping distance in *O*(*mn*) via the recurrence relation:

9 $$\begin{aligned} M_{i,j} = \min (M_{i-1,j},M_{i-1,j-1},M_{i,j-1})+ \Vert {\mathbf {x}}_{i}-{\mathbf {y}}_{j} \Vert \end{aligned}$$

The resultant $M_{m,n}$ is the dynamic time warping distance between ${\mathbf {x}}$ and ${\mathbf {y}}$. Note it is often customary to use a warping window, this limits the maximum warping that may occur between the two time series. This is trivial to implement since if TOL is our tolerance (maximum warping) then we must simply ensure that when $|i-j|>TOL$: $M_{i,j} = \infty$. By doing this we are ensuring there is an upper bound on the warping. A warping window of 0 is equivalent to Euclidean distance and this means that by considering all warping windows we are also considering Euclidean distance.

DTW is an elastic distance measure in that it measures two time series as similar even when there is misalignment along the time axis.

### Time warp edit distance

Time Warp Edit Distance (TWED) is metric that operates on time series. It was first proposed in Marteau (2008) as an elastic time series measure which unlike DTW serves as a proper metric, satisfying all the axioms of a metric function. The algorithm is both similar to DTW and to EDR. The similarity between two time series is measured as the minimum cost sequence of ”edit operations” needed to transform one time series into another. These edit operations are defined in a way which makes sense graphically. To define the “edit operations” they use the paradigm of a graphical editing process to end up with a dynamic programming algorithm that can efficiently compute TWED in roughly the same complexity as DTW.

### Edit distance on real sequences

The Edit Distance on Real sequences (EDR) distance was first published in Chen et al. (2005). The authors describe an algorithm that is an adaptation of edit distance which works on strings of letters to a distance measure that would work on time series.

The Edit Distance on Real sequences (EDR) distance was first published in Chen et al. (2005). The authors describe an algorithm that is an adaptation of edit distance which works on strings of letters to a distance measure that would work on time series.

The idea is to count the number of edit operations (insert, delete, replace) that are necessary to transform one series into the other. Of course our time series are numerical functions and therefore will almost never likely match up exactly. Therefore we use a parameter epsilon, and if two time series at a particular point in time are with epsilon of each other, we count that as a match otherwise, we don’t.

### Symbolic aggregate approximation

The approach to time series analysis above is numerical. Here we introduce a symbolic approach to time series analysis: Symbolic Aggregate Approximation (SAX). One possible motivation for moving towards a symbolic approach is that we could then utilize the wealth of datamining techniques pertaining to string representations, one example would be edit distance. Another source of motivation is that a symbolic approach may yield significant dimensionality reductions. For further explanation see Lin et al. (2007).

**Fig. 2:**
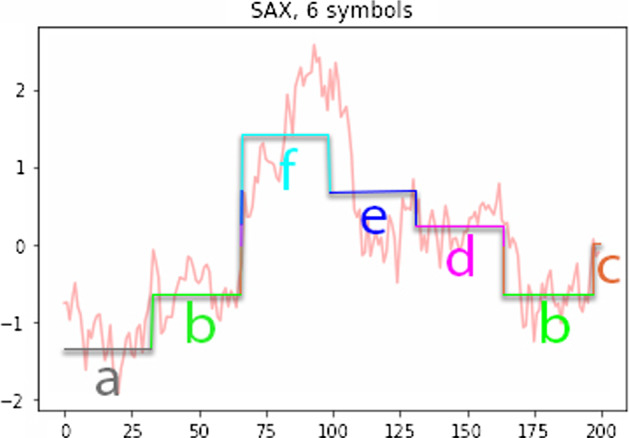
Discretization via PAA (see Mahato et al. (2018))

The fist step to SAX is to discretize the input space. First we set the alphabet size ($a>2$) for the problem. Next for every time series $C = c_{1},...,c_{n}$ we must assign each $c_{i}$ to a corresponding variable in the alphabet. So if $a=3$ and our alphabet is $\{{\mathbf {a}},{\mathbf {b}},{\mathbf {c}}\}$ then for the time series $c_{1},...,c_{n}$ we must map each $c_{i}$ to a letter in the alphabet. Our approach to discretization is to first transform the data into the Piecewise Aggregate Approximation (PAA) representation and then symbolize the PAA representation into a discrete string (Fig. 2). The two main benefits of this process are the well documented dimensionality reduction of PAA ( (Keogh et al. 2001b), (Yi and Faloutsos 2000)) and lower bounding: our distance measure between two symbolic string lower bounds the true distance between the original time series ( (Keogh et al. 2001a), (Yi and Faloutsos 2000)).

We can represent a time series *C* of length *n* in a *w*-dimensional space by a vector ${\bar{X}} = \bar{x_{1}},...,\bar{x_{n}}$. As in Lin et al. (2007), we can calculate $\bar{x_{i}}$ by

10 $$\begin{aligned} \bar{x_{i}} = \frac{w}{n}\sum \limits _{j=\frac{n}{w}(i-1)+1}^{\frac{n}{w}i}x_{j} \end{aligned}$$

We have reduced the time series from *n* (temporal) dimensions to *w* dimensions by dividing the data into *w* equally sized frames and then $\bar{x_{i}}$ is simply the mean value of the time series for that frame. We may think of this process as attempting to approximate our time series with a linear combination of box functions. It is worth noting that it is important to *z*-normalise each time series. We then appropriately define breakpoints which determine the letter in our alphabet to which each $\bar{c_{i}}$ will be mapped. Usually we do this by analyzing the statistics of our time series and choosing breakpoints so that each letter in the alphabet is as likely to appear as each other letter. In other words we choose breakpoints to spread out the data evenly. For a more thorough explanation see Lin et al. (2007). Once we have the breakpoints determined it is straightforward to map our PAA representation to a string consisting of letters from our alphabet. The PAA coefficient controls the proportion of examples that will be placed in each bin. Whereas the alphabet size regulates the discretization of the *x*-axis, the PAA coefficient regulates the discretization of the *y*-axis. When we have a large time series our approach is to first discretize the time series into a long string and then extract a bag of words. We determine a sliding window, usually found by parameter optimization, and this sliding window length becomes the length of each word in our bag of words. So we turn one long string of letters representing our original time series into a series of words, each word is the length of the sliding window. The first word starts from the first index in the original long string, the second word starts from the second index in the original long string. We proceed until all the words have been extracted.

As a distance measure between the time series we could use Euclidean distance, however to make our time series analysis more elastic we use edit distance. Now time series that are similar but not aligned will be marked as similar by our distance measure.

### Symbolic fourier approximation

Symbolic Fourier Approximation (SFA) was introduced by Schafer et al. in 2012 as an alternative method to SAX built upon the idea of dimensionality reduction by symbolic representation. Unlike SAX which works on the time domain, SFA works on the frequency domain. The algorithm is discussed and developed in Schäfer and Högqvist (2012).

SFA uses the Discrete Fourier Transform (DFT) to represent each time series as a linear combination of sines and cosines. Recall that $\{sin(kx),cos(kx)\}_{k=1}^{\infty }$ forms an orthogonal basis for real (and indeed complex) valued continuous functions. For each time series we perform what is known as an orthogonal projection onto basis functions. Let *V* be an inner product space over the field ${\mathbb {F}}$. Let ${\mathbf {v}}\in V\setminus \{0\}$. We want to decompose an arbitrary vector ${\mathbf {y}}\in V$ into the form:

11 $$\begin{aligned} {\mathbf {y}}= \alpha {\mathbf {v}}+ {\mathbf {z}}\end{aligned}$$

where ${\mathbf {z}}\in \{{\mathbf {x}}| \langle {\mathbf {x}},{\mathbf {v}}\rangle = 0 \}$ and $\alpha \in {\mathbb {F}}$. Since ${\mathbf {z}}\bot {\mathbf {v}}$ we have:

12 $$\begin{aligned} \langle {\mathbf {v}},{\mathbf {y}}\rangle = \langle {\mathbf {v}}, \alpha {\mathbf {v}}+ {\mathbf {z}}\rangle = \langle {\mathbf {v}}, \alpha {\mathbf {v}}\rangle + \langle {\mathbf {v}}, {\mathbf {z}}\rangle = \alpha \langle {\mathbf {v}}, {\mathbf {v}}\rangle \end{aligned}$$

$$\implies$$

13 $$\begin{aligned} \alpha = \frac{\langle {\mathbf {v}}, {\mathbf {y}}\rangle }{\langle {\mathbf {v}},{\mathbf {v}}\rangle } \end{aligned}$$

Whence we define the orthogonal projection of y onto v:

14 $$\begin{aligned} Proj_{{\mathbf {v}}}({\mathbf {y}}) = \frac{\langle {\mathbf {v}}, {\mathbf {y}}\rangle }{\langle {\mathbf {v}},{\mathbf {v}}\rangle } {\mathbf {v}}\end{aligned}$$

In this case our inner product is defined as:

15 $$\begin{aligned} \langle f,g \rangle = \int _{-\infty }^\infty f(x)g(x)dx \end{aligned}$$

and our basis vectors are $\mu _{k} = \{ e^{\frac{i2\pi kn}{N}}| n\in \{0,...,N-1\}\}$. If *k* were infinity it would be possible to approximate any real value continuous function to arbitrary precision in much the same was as the Taylor series can be used. However for SFA we use the discrete transformation i.e. $k < \infty$.

**Fig. 3:**
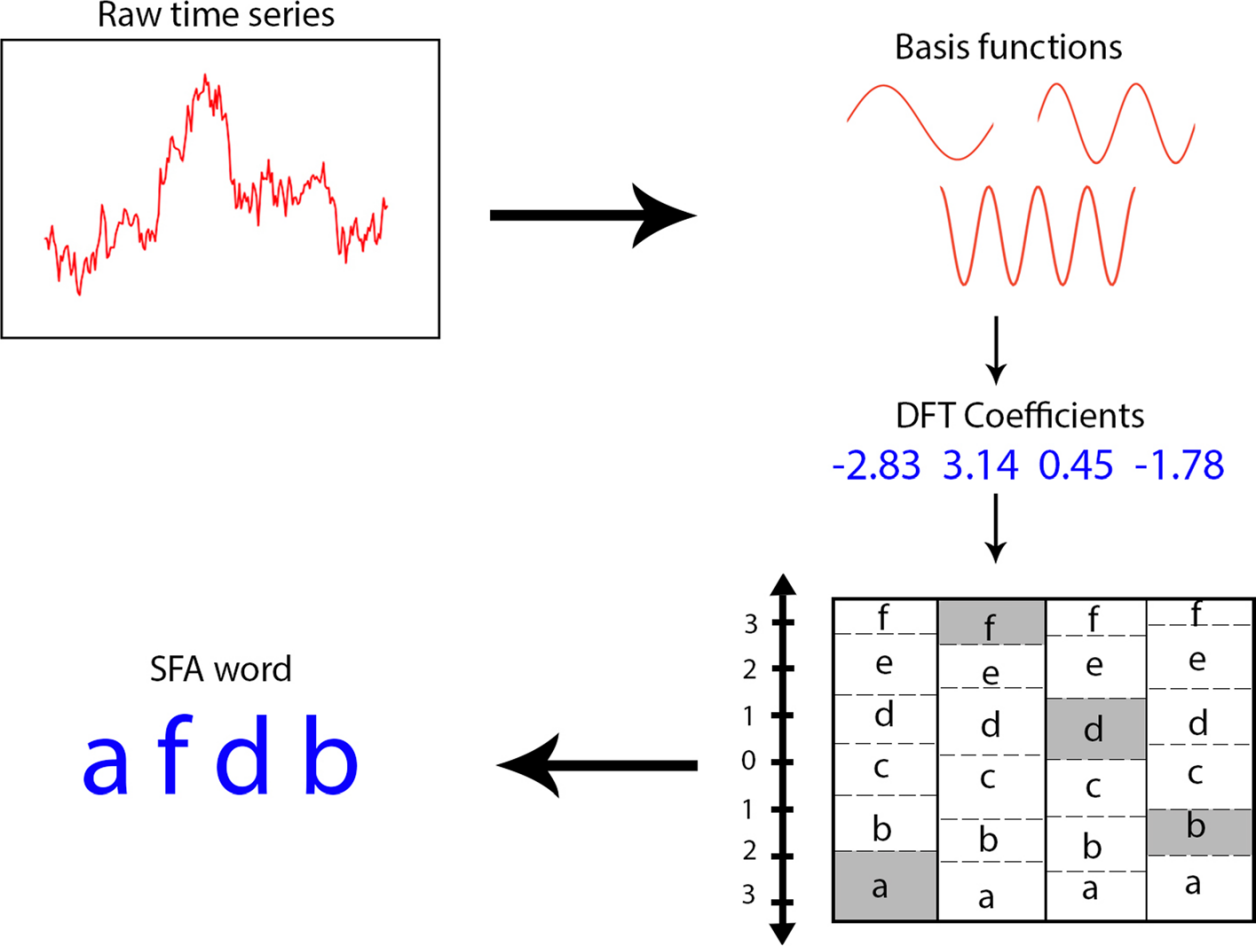
Discretization via SFA (see Mahato et al. (2018))

The DFT Approximation is a part of the preprocessing step of the SFA algorithm, where all time series data are approximated by computing DFT coefficients. When all these DFT coefficients are calculated, Multiple Coefficient Binning (MCB) is used to turn the approximated time series represented as a series of coefficients into a string representation. Next we use the very simple sliding window technique to extract a bag of features. Our first feature will be the string cut from the first letter to the length of the sliding window. Then we simply slide the window along one letter, starting from the second letter instead of the first this time. Each time we extract a feature from the string and then slide up the window by one and extract the next feature until we have exhausted the string. Once we have mapped each time series to a list of strings of length sliding window, we use edit distance to compare the string representations (Fig. 3).

## Experiments

Thirty seven datasets were selected from the UCR time series repository. We will henceforth present the results of this extensive evaluation: every distance measure presented in this paper (DTW,TWED,SAX,SFA, and EDR) paired with the Negative SVM classifier, the Gaussian SVM classifier, and the well-known kNN classifier.

Each dataset comes with a proscribed train:test split. A four-fold cross validation strategy was used and the validation set was used to optimize the parameters of the the 15 classifier-distance models via grid search. In order to ensure fairness in the parameter selection process each of the 15 classifier-distance model combinations was restricted to a grid search *budget* of 50 states. For each combination 50 distance matrices were constructed to represent 50 parameter combinations. In the case where the parameter space exceeds 50, the parameter combinations where selected from a uniform distribution. Once the optimal parameters where found, the model was build using those parameters together with the full training set and then evaluated on the held-out test set. Accuracy was calculated as expected, the ratio of correct predictions to test set size. The tables in this section present useful summaries of the results as well as all accuracy scores for each model on each dataset.

The first natural question which arises is what is the superior classifier for time series analysis. Analysing across 37 datasets, here are the average accuracies for kNN, Gaussian SVM, and Negative SVM presented in the table just below. As you can see, kNN proves to be the classifier with the highest average accuracy across all methods across all datasets (Tables 1, 2, 3, 4).

**Table 1:** Overall mean accuracy for each classifier

Classifier	Mean accuracy
kNN	0.7
Gaussian	0.6
Negative	0.59

The next overall summary would be that of the distance measure. The following table answers the question of which distance measure has the highest accuracy across all methods across all datasets.

**Table 2:** Mean accuracy of all distance measures

Distance	Mean accuracy
DTW	0.77
TWED	0.76
SAX	0.59
SFA	0.52
EDR	0.50

As you can see: DTW and TWED are the best distance measures across all datasets and across all classifiers. This is perhaps a surprising result as TWED tends to be underrated. This study finds otherwise: TWED is a very good distance measure, of almost equal efficacy to that of DTW. It is perhaps worth noting that the two distance measures are very similar.

Next, we ask which is the most successful classifier-distance pairing (model). There are two ways of approaching this questions, first we could construct a league table, where for each dataset we would award 1 point for the least accurate model and full points (at most 15) to the best performing model. Here is that league table:

**Table 3:** Classifier-distance pair league table

Model	Points
Gaussian-DTW	372
Negative-DTW	361
kNN-DTW	357
kNN-TWED	353
Negative-TWED	344
Gaussian-TWED	330
kNN-SFA	263
kNN-EDR	257
kNN-SAX	199
Gaussian-SAX	197
Negative-SAX	168
Gaussian-SFA	84
Negative-SFA	83
Gaussian-EDR	62
Negative-EDR	49

We could also simply compute the mean accuracy of each model across all the datasets:

**Table 4:** Mean accuracy of all models

Classifier	Distance	Mean accuracy
Gaussian	DTW	0.78
Negative	DTW	0.77
Negative	TWED	0.77
kNN	DTW	0.76
kNN	TWED	0.76
Gaussian	TWED	0.76
kNN	EDR	0.68
kNN	SFA	0.68
kNN	SAX	0.62
Gaussian	SAX	0.60
Negative	SAX	0.55
Negative	SFA	0.44
Gaussian	SFA	0.44
Gaussian	EDR	0.41
Negative	EDR	0.39

Both tables agree that the best model for time series classification is the Gaussian-SVM paired with the DTW distance measure.

You can think of the latter table as a goal difference score, whereas the former table is the league points, two different measures of performance which both agree that Gaussian-DTW is the superior model amongst all here studied.

Finally, here are three tables documenting the results of each experiment conducted (over 400 in total) (Tables 5, 6).

**Table 5:** The kNN classifier evaluated on all 37 datasets. Figures in bold indicate the best results for that dataset

Dataset	DTW	TWED	EDR	SAX	SFA
Birdchicken	0.6	0.6	0.7	0.45	**0.8**
Beetlefly	0.65	0.75	0.6	0.6	**0.85**
Coffee	**0.96**	0.93	**0.96**	0.89	0.75
Beef	**0.63**	0.4	0.4	0.27	0.53
Oliveoil	0.77	0.8	0.17	0.8	**0.9**
Wine	**0.65**	0.59	0.43	**0.65**	0.5
Facefour	0.9	**0.94**	0.91	0.3	0.59
Meat	**0.93**	0.92	0.67	0.83	0.72
Car	0.72	**0.73**	0.72	0.63	0.48
Lighting2	**0.8**	0.75	0.74	0.61	0.7
Herring	0.59	0.53	0.5	**0.62**	0.59
Lighting7	0.71	**0.74**	0.64	0.4	0.44
Toe segmentation2	0.85	0.76	**0.87**	0.73	0.41
Trace	**0.99**	0.93	0.77	0.65	0.96
ECG200	0.89	**0.9**	0.89	0.8	0.82
Shapelet sim	0.71	**0.82**	0.48	0.46	0.46
Gun point	0.96	**0.99**	0.97	0.97	0.85
Plane	**1.0**	0.99	0.98	0.99	0.94
Arrow head	**0.79**	0.72	0.66	0.43	0.75
Ham	**0.67**	0.65	0.55	0.56	0.64
Worms two class	**0.68**	0.63	0.61	0.44	0.62
Worms	**0.5**	0.47	0.36	0.4	0.46
ToeSegmentation1	0.79	**0.81**	0.59	0.55	0.62
Diatom size reduction	**0.93**	**0.93**	0.92	0.79	0.73
FISH	0.85	**0.93**	0.76	0.58	0.65
OSU leaf	0.58	**0.72**	0.48	0.37	0.6
Earthquakes	**0.82**	**0.82**	0.8	0.81	0.79
Haptics	**0.41**	**0.41**	**0.41**	0.24	0.3
Computers	0.62	**0.72**	0.68	0.64	0.68
Distal phalanx outline age group	**0.84**	**0.84**	0.82	0.75	0.77
Distal phalanxTW	**0.79**	0.74	0.74	0.68	0.76
Middle phalanxTW	0.61	**0.63**	0.61	0.58	0.61
Middle phalanx outline age group	**0.79**	**0.79**	**0.79**	0.66	0.71
Synthetic control	**0.97**	0.96	0.83	0.68	0.79
Proximal phalanxTW	**0.81**	0.8	0.8	0.76	0.78
Proximal phalanx outline age group	0.84	0.84	0.83	**0.85**	0.81
Sony AIBO robot surface	0.69	0.69	0.69	0.58	**0.75**
Mean	**0.76**	**0.76**	0.68	0.62	0.68

As you can see, for kNN, the most accurate distance measures are DTW, and TWED.

**Table 6:** The Gaussian SVM evaluated on 37 datasets. Figures in bold indicate the best results for that dataset

Dataset	DTW	TWED	EDR	SAX	SFA
Bird chicken	0.65	0.7	0.5	**0.85**	0.5
Beetlefly	**0.7**	0.65	0.5	0.45	0.45
Coffee	**1.0**	**1.0**	0.54	0.61	0.54
Beef	**0.67**	0.63	0.2	0.6	0.2
Oliveoil	**0.87**	**0.87**	0.4	**0.87**	0.4
Wine	0.78	0.74	0.5	**0.81**	0.5
Facefour	**0.89**	0.86	0.16	0.36	0.48
Meat	**0.97**	0.93	0.33	0.83	0.33
Car	0.63	**0.68**	0.22	0.38	0.22
Lighting2	0.72	**0.75**	0.54	0.56	0.54
Herring	0.53	**0.59**	**0.59**	**0.59**	**0.59**
Lighting7	**0.84**	0.73	0.26	0.26	0.26
Toesegmentation2	0.81	**0.85**	0.82	0.53	0.82
Trace	**0.99**	0.89	0.19	0.62	0.19
ECG200	0.75	**0.93**	0.64	0.71	0.85
Shapelet sim	**0.79**	0.46	0.5	0.5	0.5
Gun point	0.92	**0.98**	0.49	0.89	0.49
Plane	**1.0**	0.97	0.1	0.99	0.1
Arrow head	0.74	**0.76**	0.39	0.46	0.39
Ham	**0.71**	**0.71**	0.51	0.63	0.51
Worms two class	**0.61**	0.58	0.58	0.58	0.58
Worms	**0.46**	0.42	0.42	0.42	0.42
Toe segmentation1	**0.86**	0.76	0.53	0.64	0.55
Diatom size reduction	**0.93**	0.92	0.3	0.3	0.3
FISH	**0.87**	0.83	0.13	0.55	0.13
OSUleaf	**0.59**	0.52	0.18	0.23	0.18
Earthquakes	**0.82**	**0.82**	**0.82**	**0.82**	**0.82**
Haptics	0.43	**0.47**	0.21	0.39	0.21
Computers	**0.68**	**0.68**	0.5	0.59	0.6
DistalPhalanx outline age group	**0.86**	0.81	0.64	0.81	0.64
DistalPhalanxTW	**0.79**	0.77	0.53	0.74	0.53
Middle phalanxTW	0.61	**0.62**	0.21	0.6	0.21
Middle phalanx outline age group	0.78	**0.79**	0.27	0.6	0.27
Synthetic control	**0.98**	0.97	0.17	0.27	0.17
Proximal phalanxTW	**0.81**	**0.81**	0.45	0.78	0.79
Proximal phalanx outline age group	**0.86**	0.85	0.49	0.84	0.49
SonyAIBO robot surface	**0.79**	0.74	0.43	0.63	0.43
Mean	**0.78**	0.76	0.41	0.6	0.44

As you can see, again for the Gaussian kernel, DTW and TWED are the most accurate distance measures (Tables 7, 8).

**Table 7:** The negative SVM evaluated on all 37 datasets

dataset	DTW	TWED	EDR	SAX	SFA
Bird chicken	**0.8**	0.7	0.5	0.6	0.5
Beetlefly	0.75	**0.85**	0.5	0.45	0.75
Coffee	**1.0**	**1.0**	0.46	0.43	0.46
Beef	0.67	**0.73**	0.2	0.67	0.2
Olive oil	**0.87**	**0.87**	0.4	0.4	0.4
Wine	0.76	**0.87**	0.5	0.57	0.5
Face four	**0.89**	0.86	0.3	0.32	0.41
Meat	**0.97**	0.93	0.33	0.8	0.33
Car	0.58	**0.8**	0.22	0.22	0.22
Lighting2	0.66	**0.75**	0.54	0.54	0.54
Herring	**0.59**	**0.59**	**0.59**	**0.59**	**0.59**
Lighting7	**0.89**	0.74	0.26	0.33	0.38
Toe segmentation2	**0.81**	0.75	0.18	0.73	0.54
Trace	**1.0**	0.9	0.19	0.64	0.19
ECG200	0.72	**0.92**	0.64	0.64	0.82
ShapeletSim	**0.82**	0.48	0.5	0.49	0.5
Gun point	0.85	**0.97**	0.49	0.87	0.49
Plane	**1.0**	0.98	0.1	0.89	0.1
Arrow head	0.74	**0.77**	0.3	0.34	0.53
Ham	0.71	**0.72**	0.51	0.46	0.51
Worms two class	0.61	0.58	0.58	**0.64**	0.58
Worms	0.44	0.42	0.42	**0.45**	0.42
Toe segmentation1	**0.84**	0.6	0.47	0.55	0.47
Diatom size reduction	**0.96**	0.93	0.3	0.73	0.59
FISH	**0.86**	0.81	0.13	0.24	0.13
OSULeaf	**0.51**	0.5	0.18	0.31	0.18
Earthquakes	**0.82**	**0.82**	**0.82**	**0.82**	**0.82**
Haptics	0.29	**0.45**	0.21	0.33	0.21
Computers	0.57	**0.68**	0.5	0.61	0.54
DistalPhalanx outline age group	**0.86**	0.81	0.64	0.81	0.74
DistalPhalanxTW	**0.79**	0.77	0.53	0.74	0.53
MiddlePhalanxTW	0.6	**0.64**	0.21	0.61	0.21
MiddlePhalanx outline age group	**0.8**	0.77	0.27	0.27	0.27
Synthetic control	**0.98**	0.96	0.17	0.19	0.17
ProximalPhalanxTW	0.81	**0.82**	0.45	0.78	0.45
ProximalPhalanx outline age group	0.86	0.85	0.49	**0.87**	0.49
SonyAIBO robot surface	**0.79**	0.71	0.43	0.43	0.43
Mean	**0.77**	0.76	0.39	0.55	0.44

Finally, once again, for the Negative Kernel, DTW and TWED are tied as the best distance measure.

### Computational cost reduction

When using a Support Vector Machine, all training samples that are not support vectors may be discarded. This produces a reduction in computational cost. We investigated the mean support vector density (ratio of support vectors to training samples) and display the results in the following table.

**Table 8:** Support vector densities

Classifier	Distance	Mean	Standard deviation
Negative	DTW	0.77	0.16
Gaussian	DTW	0.83	0.16
Negative	TWED	0.86	0.13
Gaussian	TWED	0.89	0.12
Gaussian	EDR	0.91	0.13
Negative	EDR	0.91	0.13
Gaussian	SAX	0.91	0.14
Gaussian	SFA	0.91	0.13
Negative	SAX	0.92	0.14
Negative	SFA	0.93	0.11

**Fig. 4:**
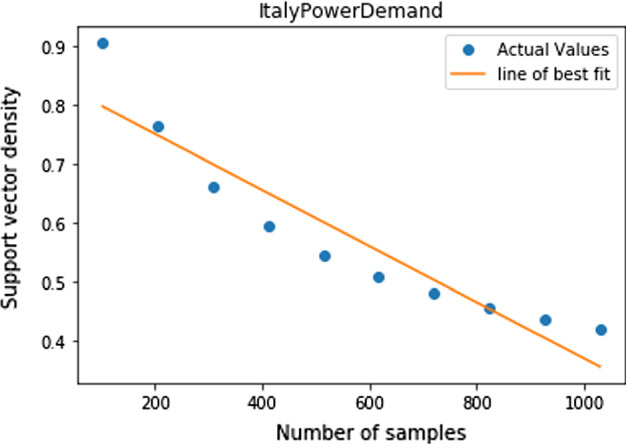
Support vector density against subset size for the Italy power demand dataset

As you can see, the Negative-DTW model produces the greatest saving in computational cost on average. However, upon further investigation we found that for this model the saving tends to increase as samples are added and decreases as time series length is decreased, a completely intuitive results. The correlation coefficients between training set size and support vector density; and training set size and time series length where -0.27 and 0.11 respectively, confirming that deduction. Finally, to complete our reduction investigation and to confirm this intuitive result, we computed a Negative-DTW kernel matrix for a much larger dataset the ”ItalyPowerDemand” dataset which has 1029 training samples of length 24. The support vector density was very low at only 42%. This means that only 430 of the 1029 training samples would be needed in model prediction. A massive saving in computational cost seeming to confirm the hypothesis that given many samples of relatively short length, the reduction in computational cost derived from using a support vector will be substantial. To validate this theory, we selected random subsets of the training set and plotted the support vector density of that subset against the size of the subset in Fig. 4.

We must state that technique exist for kNN-DTW models to reduce computational cost too, by bounding techniques. However, these work on best on long time series, and have little or no effect at all on shorter time series. On the other hand, the SVM does the opposite working well for shorter time series, especially when there is a large training set size relative to the length of the time series.

We also ran various weighted league tables, using time series length or dataset size as weights, the results remained unchanged. Gaussian-DTW remains the best model even after weightings.

The two best algorithms found in these experiments are not without flaw. The principle flaw is the same for both, it is their global operation. Both algorithms consider the whole time series, however if a small subsample of the time series is crucial and highly predictive whereas the rest has little predictive power, then the predictive power of the subsample would be diluted by the remaining time series. This problem can be tackled by taking a slice of all the time series and using these slices as inputs into the distance algorithms. We should then repeat this taking a different slice of the time series each time, until we have found the optimal slice with respect to the validation set. Another solution would be to use motifs (Yeh et al. 2017) which work by finding a pattern in the time series which is highly predictive.

SAX and SFA have a very large parameter space and this is a major limitation. To tackle this we used a random grid search, which is a grid search where parameter values are chosen at random from within the grid. Better heuristics must be developed to guide or replace grid search in finding the optimal parameters. From examining a very large number of cross-validation train accuracy scores it seems that very often small variations in parameters will not affect accuracy and hence there may be redundancy in the parameters, or at the very least, we can say that there is definitely the possibility of automating (or improving the automation of) the setting of certain parameters. For example, if we know that a given combination of parameters has an extremely low accuracy, perhaps that means that we need not search for parameter points in the near vicinity. It is perhaps worth noting that if this is indeed a fact, Particle Swarm Optimization would be an excellent choice for an intelligent stochastic optimization algorithm in order to search through parameters, the cross-validation accuracy acting as the objective function to be maximized.

## Conclusions

In this paper we have presented experiments with the hopes of identifying time series distance-kernel pairs which provide the highest accuracy. We have found two superior distance-kernel pairs: Gaussian-DTW and Negative-DTW, with Gaussian-DTW having the slight edge over its primary rival. KNN is found to be a robust classifier measure compatible with all distances studies, while DTW and TWED are found to be robust distance measures compatible with all classifiers studied. However, this paper identifies Gaussian-DTW as the best model for time series classfication.

There is an advantage of using an SVM over using kNN and that is the saving that results from discarding training samples. Every training sample that is not a support vector is not used for model prediction. We have found that the Negative-DTW on average retains 77% of samples whereas Gaussian-DTW retains 83% of samples. This saving is marginal, however upon analysis we identified that the support vector density tends to decrease as the size of the training set increases and tends to increase as the length of the time series in the dataset increases. The correlation coefficient in both cases easily lead us to this deduction, and so there is great merit in using an SVM.

A major finding of this paper is that the most reliable distance measure for use in a Gaussian-SVM is Time Warp Edit Distance, as although it is marginally less accurate than DTW, it is guaranteed to generate a positive semi-definite kernel matrix on every dataset, which is a major advantage when it comes to model reliability.

Finally, we reflect on the inferior distance measures. For EDR there can be no excuses, EDR is simply poorly adjusted to time series classification. For SAX and SFA we may make some excuses. Their parameter space is massive so the restriction to fifty randomly selected parameter combination may significantly interfere with their performance, and a relaxation of this restriction would most likely improve their performance. However, the restriction is reasonable in a fair competition. Perhaps the inventors of those distance measures should investigate the parameters further and attempt to identify heuristics which would allow for a better than random selection from the very large parameter space.

In summation and conclusion, we have found that Support Vector Machines are a state-of-the-art classifier for time series and are indeed very useful in the domain of time series analysis. The Gaussian-DTW SVM outperforms 1NN-DTW, the assumed baseline for time series, and is a better classifier. Not only that, but using an SVM instead of a kNN model results in a significant reduction in computational cost when there are many training samples relative to time series length. We can therefore boldly recommend the Gaussian-DTW SVM for time series classification.

In future we will aim to determine heuristics that allow us to combine the output from the best distance measures examined in this body of work in order to produce a more robust distance measure, one which is an optimal combination of the composite kernels. This will inevitably lead to an attempt at adapting Multiple Kernel Learning techniques to time series analysis. We also plan to move on from the univariate time series analysed here to research multivariate time series in order to see if DTW and TWED will also be superior in those scenarios.

## Footnotes

1 The triangle inequality which is a necessary condition for the distance measure *d* on a vector space $\chi$ to be a metric states: $d(u,v)\le d(u,w)+d(w,u) \quad \forall u,v,w \in \chi$ i.e. the shortest distance between two points is the straight line (direct route) connecting them.
